# Sociodemographic Predictors of Health Risk Perception, Attitude and Behavior Practices Associated with Health-Emergency Disaster Risk Management for Biological Hazards: The Case of COVID-19 Pandemic in Hong Kong, SAR China

**DOI:** 10.3390/ijerph17113869

**Published:** 2020-05-29

**Authors:** Emily Ying Yang Chan, Zhe Huang, Eugene Siu Kai Lo, Kevin Kei Ching Hung, Eliza Lai Yi Wong, Samuel Yeung Shan Wong

**Affiliations:** 1 Collaborating Centre for Oxford University and CUHK for Disaster and Medical Humanitarian Response (CCOUC), The Chinese University of Hong Kong, Hong Kong, China; huangzhe@cuhk.edu.hk (Z.H.); euglsk@cuhk.edu.hk (E.S.K.L.); kevin.hung@cuhk.edu.hk (K.K.C.H.); 2 Nuffield Department of Medicine, University of Oxford, Oxford OX37BN, UK; 3 JC School of Public Health and Primary Care, The Chinese University of Hong Kong, Hong Kong, China; lywong@cuhk.edu.hk (E.L.Y.W.); yeungshanwong@cuhk.edu.hk (S.Y.S.W.); 4 Accident & Emergency Medicine Academic Unit, The Chinese University of Hong Kong, Prince of Wales Hospital, Hong Kong, China

**Keywords:** COVID-19, urban, health risks, Health-Emergency and Disaster Risk Management, biological hazard, pandemic, PHEIC, Hong Kong

## Abstract

In addition to top-down Health-Emergency and Disaster Risk Management (Health-EDRM) efforts, bottom-up individual and household measures are crucial for prevention and emergency response of the COVID-19 pandemic, a Public Health Emergency of International Concern (PHEIC). There is limited scientific evidence of the knowledge, perception, attitude and behavior patterns of the urban population. A computerized randomized digital dialing, cross-sectional, population landline-based telephone survey was conducted from 22 March to 1 April 2020 in Hong Kong Special Administrative Region, China. Data were collected for socio-demographic characteristics, knowledge, attitude and risk perception, and various self-reported Health-EDRM behavior patterns associated with COVID-19. The final study sample was 765. Although the respondents thought that individuals (68.6%) had similar responsibilities as government (67.5%) in infection control, less than 50% had sufficient health risk management knowledge to safeguard health and well-being. Among the examined Health-EDRM measures, significant differences were found between attitude and practice in regards to washing hands with soap, ordering takeaways, wearing masks, avoidance of visiting public places or using public transport, and travel avoidance to COVID-19-confirmed regions. Logistic regression indicated that the elderly were less likely to worry about infection with COVID-19. Compared to personal and household hygiene practices, lower compliance was found for public social distancing.

## 1. Introduction

The COVID-19 pandemic was considered a Public Health Emergency of International Concern (PHEIC) by the World Health Organization (WHO) on 30 January 2020 [1]. The SARS-CoV2 belongs to the coronavirus family, which is the same family as the severe acute respiratory syndrome (SARS) and middle east respiratory syndrome (MERS) viruses [2]. It was first reported as a cluster of respiratory illnesses in Wuhan, China, on 30 December 2019. As of 12 April 2020, there were 1,844,863 confirmed cases and 117,021 deaths according to the World Health Organization report [3]. The case-fatality rate resulting from SARS-CoV2 is believed to be around 1%–2% for symptomatic cases [4,5], and the proportion of asymptomatic cases of COVID-19 is much higher [6]. With a higher basic reproductive number than SARS and MERS, and the finding of viral shedding in asymptomatic patients [7], the total number of people infected and killed by COVID-19 exceeds SARS and MERS combined, even though it is less deadly than them [8]. As of April 2020, although more than 200 countries have reported confirmed cases and have implemented travel restrictions [9], social distancing [10] and other response measures to this PHEIC, the global COVID-19 epidemic is yet to end.

### The Situation in Hong Kong and Past Experiences of Similar Pandemics

**Figure 1:**
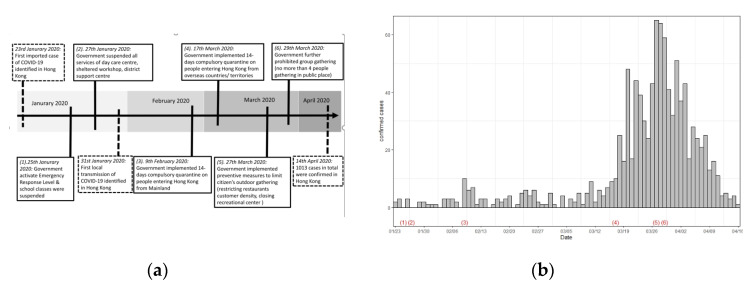
(**a**) Timeline of the COVID-19 outbreak in Hong Kong and the preventive measures implemented by the Hong Kong government. (**b**) The confirmed cases in Hong Kong from 23 January 2020 to 16 April 2020. Note: (1) to (6) in Figure 1b correspond to (1) to (6) in Figure 1a.

Figure 1 shows that on 23 January 2020, Hong Kong reported its first imported case of COVID-19 [11] and the first local transmission emerged on 31 January 2020 [12]. As of 14 April, there were 1013 confirmed cases [13]. As soon as cases were confirmed in Hong Kong, the Hong Kong government implemented various infection control measures (Figure 1a), including declaring emergency response levels in relation to COVID-19 infection [14], suspending class resumption for all schools and community services [15,16], and prohibiting citizen outdoor activities and gatherings [17,18,19,20]. At that time, there were a substantial number of confirmed cases of the epidemic (Figure 1b).

The Health-Emergency and Disaster Risk Management (Health-EDRM) Framework provides a common language and a comprehensive approach for all actors in health and other sectors, to reduce health risks and consequences of emergencies and disasters [21]. Biological hazards, such as infectious disease outbreaks, are considered one of the major health risks for the human population. Besides top-down government efforts in infection control and management, efforts at the individual and household level also have crucial roles in bottom-up resilience of Health-EDRM for biological hazards. Currently, limited scientific evidence is available to understand patterns of knowledge, perception, attitude and behavior undertaken by urban populations for relevant disaster risk reduction programs and policy planning. Hong Kong, as an Asian metropolis in Southern China, has encountered various infectious disease outbreaks, like SARS [22] and avian influenza (e.g., H5N1 and H7N9) [23,24]. Its healthcare system has pre-existing policies and practices against emerging infectious diseases [25]. For example, wearing face masks, washing hands, and disinfecting living quarters for SARS [26], and avoiding contact with birds for the avian influenza [27], are examples of community bottom-up Health-EDRM practices for self-help and contribution to infection control. This study, using a population-based, computerized randomized digital dialing landline telephone survey, intends to examine and explore various primary Health-EDRM prevention efforts in the community through self-reported knowledge, perception and behaviors related to COVID-19 and various droplet borne-transmission control-related Health-EDRM preventive measures among the Hong Kong population [28,29].

## 2. Materials and Methods

### 2.1. Study Design and Study Population

A computerized randomized digital dialing (RDD), cross-sectional, population landline-based telephone survey was conducted from 22 March to 1 April 2020. The study population consisted of Hong Kong residents aged 18 years or above, including those holding valid work or study visas. Sample exclusion criteria included (i) non-Cantonese-speaking respondents; (ii) overseas visitors holding tourist visas to Hong Kong or two-way permit holders from mainland China; (iii) those unable to be interviewed due to medical reasons; and iv) non-institutional residents. For the sample size estimation, an assumption was made that 50% of the Hong Kong population were concerned about contracting COVID-19. A sample size of 750 participants was calculated with a 3.6% margin of error and 95% confidence interval.

### 2.2. Data Collection

**Figure 2:**
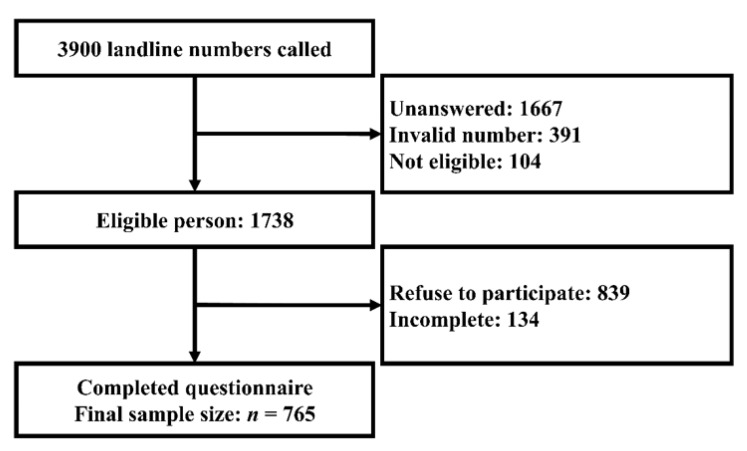
The algorithm of the final data collection.

The computerized random digit dialing (RDD) method was used for each of Hong Kong’s 18 districts to randomly select a representative sample. Stratified random sampling was used to ensure the demographic representation of the general population in Hong Kong in terms of age, gender, and district of residence. This data collection method has been used for other similar local studies on infectious diseases [30,31]. Most of the calls were made during evenings (6:30 pm to 10:00 pm) to avoid an under-representation of the working population. An eligible family member whose birthday was closest to the survey date was invited to participate in the study. If the selected participant was busy at the time, up to four follow-up calls would be made before that number was considered unanswered. All telephone interviews were conducted by trained interviewers in Cantonese. Figure 2 shows that 765 eligible participants were recruited to account for missing values and increase the modelling flexibility.

### 2.3. Study Instrument

A self-reported structured Chinese questionnaire with 141 questions was designed on the basis of a literature review and previous research experience [27,30,31,32,33,34] on data collection. Information was collected on socio-demographic characteristics, knowledge, attitude and risk perception, and various behavior patterns associated with COVID-19. Sociodemographic information, current and preferred channels of information acquisitions were similar to a number of published study tools of human behavior in extreme events in the same context [30,35,36]. Questions related to “knowledge about COVID-19”, “risk perception”, “self-reported perception and Health-EDRM practices” and “caregiving”, were adopted from previous studies [27,30,31]. A summary of the survey questions can be grouped into six major subgroups as follows.

1. Sociodemographic information was collected for age, gender, district of residence, household income, household size, marital status, education, size of housing, occupation and employment status.
2. Knowledge about COVID-19, including the transmission route, and the comparison between COVID-19 and other respiratory diseases.
3. Risk perception of Health-EDRM behavior associated with COVID-19, including the perceived impacts (e.g., physical, mental, social, financial and the whole impact), perceived sufficient knowledge to manage COVID-19, perceived severity and infectivity. Five-point Likert scales were used to measure the level of agreement or disagreement for the questions (from 1 to 5, 1 = strongly disagree, 2 = disagree, 3 = neutral, 4 = agree, 5 = strongly agree). The 6-item short form of the State-Trait Anxiety Inventory (STAI) was used for measuring their current anxiety level concerning the outbreak [32]. A binary question of whether the respondent was worried about getting infected with COVID-19 was asked.
4. Self-reported perceived usefulness and actual Health-EDRM behavioral practice of nine personal or household health emergency disaster risks management related behaviors and practices of COVID-19 prevention behavior. These include: (1) washing hands before meals and after toileting, (2) washing hands with soaps, (3) avoiding dining or gathering together, (4) using serving utensils, (5) ordering takeaways more often, (6) bringing one’s own utensil when dining out, (7) wearing a mask when going out, (8) avoiding going to public places or using public transport, and (9) avoiding going to COVID-19-confirmed regions outside Hong Kong. The four-point Likert scale was used to ascertain the level of the practices (from 1 to 4, 1 = always, 2 = usually, 3 = sometimes, 4 = never).
5. Current and preferred channels of information acquisition, the information they were interested in and the awareness of COVID-19.
6. Questions about home quarantine and caregiving to non-suspected family members during the COVID-19 outbreak were also asked.

We focused on the general patterns of health risk perception, attitude and behavior practices associated with Health-Emergency Disaster Risk Management. Each interview took about 20–40 min. A pilot survey study (*n* = 28) was conducted in March 2020 to ensure interpretability and feasibility of the questions. Verbal consent was obtained from the participants and ethics approval and the consent procedure of the study were reviewed and obtained from the Survey and Behavioral Research Ethics Committee at The Chinese University of Hong Kong (SBRE-19-498).

### 2.4. Statistical Analysis

Descriptive statistics were reported for the study sample socio-demographic characteristics, awareness, perception and knowledge of COVID-19. Statistical association tests (Pearson’s χ^2^ test, Fisher’s exact test, or McNemar’s test) were conducted where appropriate. A binary variable of whether the respondent was worried about getting infected with COVID-19 was used as the dependent variable in logistic regression. Explanatory variables entered into multivariable logistic regression if the *p*-value < 0.10 in bivariate analysis. Apart from the worry, various community patterns and individual uptake of Health-EDRM behavior associated with COVID-19 as dependent variables were dichotomized for logistic regression (“always” or “usually” versus “sometimes” or “never”). The level of significance of the statistical test was 0.05. All statistical analyses were conducted by using IBM SPSS 21 (IBM Corp., Armonk, NY, USA) for Windows.

## 3. Results

The final study sample consisted of 765 respondents and a response rate of 44.0% (765/1738) (Table 1). The final study sample was comparable and representative of the population data in the Hong Kong Census 2016 [37] with respect to their age, gender, marital status and residential districts. However, the level of education and household income in our sample were higher than the population census.

### 3.1. Perception of Various Health and Economic Impacts of COVID-19

**Figure 3:**
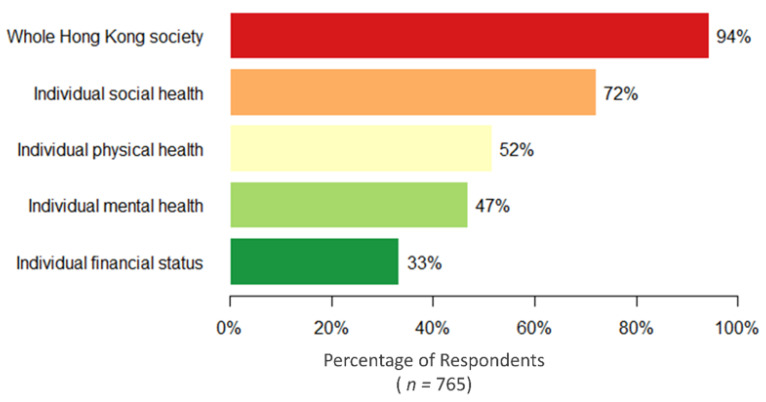
Self-reported large or very large impacts of COVID-19 on various dimensions.

Figure 3 describes the respondents’ perception of various health impacts brought on by COVID-19. Although 94.4% (722/765) of the study respondents believed that COVID-19 had a large impact on their community, social health was reported as the most affected (72.0%). In addition, participants reported the role of government policy (68.6%) would be similar to the effort put in at the household or individual level (67.5%). However, although 63.9% (489/765) believed they had enough knowledge for regular communicable diseases (e.g., influenza), less than half of the participants (47.8%) reported that they had sufficient knowledge to manage the health risk and safety during the outbreak of COVID-19. After adjusting for age, gender, education, household income, and occupation, multiple logistic regression suggested that people aged 65 or above were less likely to be impacted by COVID-19 in terms of their mental, social and financial status, while people with less household income were more likely to be affected financially. In addition, females were more likely to report a large impact on their mental health, but none of the above sociodemographic variables were associated with the reported physical health (Appendix A Table A1).

### 3.2. Knowledge and Risk Perception of COVID-19

**Figure 4:**
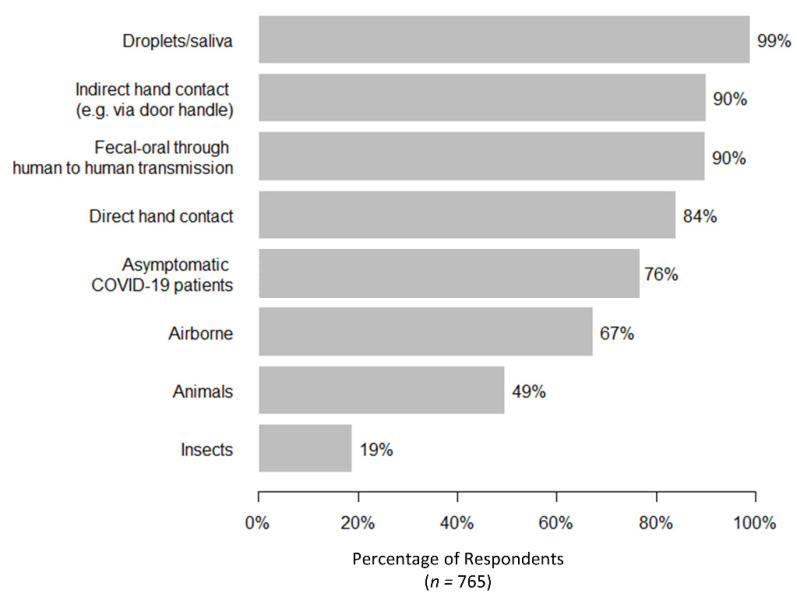
Reported believed transmission mode for COVID-19.

Regarding the overall knowledge and understanding of COVID-19, results indicated that most respondents could identify that the disease could be transmitted through droplets, direct or indirect hand contact, fecal contamination, and contact with asymptomatic patients (Figure 4). Yet, confusion was found with some reporting of unconfirmed transmission routes (e.g., insects as vectors) and about 24% (181/764) of the respondents did not believe that asymptomatic patients could transmit the disease, which might affect adoption of appropriate practices. Respondents with a higher level of education were more likely to correctly identify whether insects and asymptomatic patients can transmit the virus (Appendix A Table A1).

For the perceived infectivity of COVID-19, about 96% (34/765) believed the infectivity was high or very high and that it was much higher than SARS (78.0%) and seasonal influenza (52.5%). For the perceived severity, about 80% (156/765) believed it had a severe or very severe harm to health, which was less than SARS (90.5%) but higher than seasonal influenza (21.6%).

### 3.3. Attitude and Uptake of Health-EDRM Behavior Practice towards COVID-19

Although the uptake of Health-EDRM practice varied, most respondents agreed that personal or household preventive measures could reduce the transmission of COVID-19 (Table 2). Significant statistical differences were found between attitude and practice in regards to washing hands with soap, ordering takeaways more often, wearing masks when going out, avoidance of visiting public places or using public transport, and avoidance of travelling beyond COVID-19-confirmed regions outside Hong Kong. Furthermore, a comparison of the behavior of wearing masks before and after the epidemic found that when going outdoors, mask wearing had increased from 11.3% (86/764) before the epidemic to 97.4% after the disease outbreak (McNemar’s test, *p*-value < 0.001). Compared to personal and household hygiene, compliance of social distancing in public was lower. After adjusting for age, gender, education, household income, and occupation, multiple regression revealed that male gender was significantly negatively associated with the behavior of washing hands with soap and the avoidance of dining and gathering together. Compared to white collar workers, housewives and the unemployed or retired were more likely to avoid going to public places or using public transport (Appendix A Table A2).

Around 32.7% (*n* = 249) of respondents believed in religion. Among them, analysis of mass gathering activity showed that 68.7% (171/249) reported that they reduced their religious gathering behavior during this pandemic. In addition, about a quarter of the study population (*n* = 181) reported that they had traveled abroad since January 2020. Mainland China (30.6%) and Japan (25.0%) were the most popular travelling areas.

### 3.4. Sociodemographic Factors Affecting Anxiety around Getting COVID-19

Among all the respondents, 66.7% (252/757) worried that they would become infected with COVID-19 with a mean STAI score of 2.57. Bivariate analyses of different socio-demographics, perception of the effect of COVID-19, and perceived infectivity and severity towards the worry about getting COVID-19 were conducted (Table 3). A multivariable logistic regression (Omnibus tests of model coefficients chi-square: 86.9, df = 10) revealed that young age, respondents who perceived significant COVID-19 impact on their physical, mental health, and/or social life were more likely to express anxiety of being infected. In addition, as the first global metropolis to report house pet SARS-CoV2 infection, our study showed that among the 124 respondents who are house pet owners, 18.5% (23/124) worried that their pets will also be infected with COVID-19.

The multivariable regression revealed that except for age, sociodemographic factors including gender, chronic disease status, education, marital status, household income, household floor area and district of residence were not significantly associated with the concern of becoming infected, which suggested that the worry of COVID-19 was indiscriminate across different sociodemographic factors in Hong Kong. Meanwhile, believing COVID-19 had a large effect on their physical, mental or social health was more likely to cause worry that they will be infected.

### 3.5. Other Related Behavioral Experiences: Home Quarantine and Caregiving to Non-Infected Family Members during COVID-19

Of the study respondents, 4.2% (32/765) reported that they practiced home-quarantine for COVID-19, where 71.9% (23/32) were volunteers and 28.1% (9/32) were compulsory quarantined. Most cited reasons for home-quarantine was history of recent travel abroad (13/32 = 40.6%) and close contact with confirmed patients (6/32 = 18.8%). About 83.8% subjects believed that quarantine was effective in infection control. During the COVID-19 epidemic, 25.1% of respondents (*n* = 192) reported that they engage in regular home and social care responsibilities. Among all the care providers, around 20% reported that they previously used community services and centers (e.g., school and day care centers) before COVID-19. Meanwhile, among these community service users, about 40% had stopped or decreased the use of those services due to closure during the epidemic. Respondents reported the need to take care of one (45.8%) or two family members (35.4%). About 28% and 7.4% of these respondents had been caring for frail older adults and those with disabilities, respectively. More details on caregiving to non-infected family members will be reported separately.

### 3.6. Information Channel and Type of Information of Health Information Seeking for COVID-19

More than 95% of the respondents reported that they were continuously concerned about the development of the COVID-19 epidemic. The main reported information seeking channels were television (36.1%), internet (28.8%) and smartphone apps (27.6%). When asked what kind of information they wanted to know, 88.3% and 87.7% of respondents wanted to know information about a vaccine and the situation of the epidemic, respectively.

## 4. Discussion

Using the standard computerized RDD method, this population landline-based study showed the self-reported perceived health impact, and the health emergency and disaster risk management (Health-EDRM) related preventive measures uptake (both individual or household level, and government level) against COVID-19 among the Hong Kong population. Consistent with other telephone survey results at early stages of the pandemic reported in Hong Kong, most respondents continued to report high perceived severity of COVID-19 [38]. In addition, a higher anxiety level during COVID-19 (STAI of 2.57) was seen in this study when compared with previous studies using the comparable scales conducted during SARS (2.24) in 2003 [39], and the H7N9 epidemic (1.85) in 2014 [27].

For various health impacts on demographic subgroups, people aged 65 or above reported that they were less affected by COVID-19 in terms of mental and social health, and these factors were also found to be significantly associated with concern of COVID-19. Findings were consistent with the online survey study results in mainland China [40] that also reported a higher anxiety level among younger respondents. The previous published online survey held in Hong Kong in February 2020 [41] had reported a higher proportion of respondents in distress from COVID-19, which might be explained by the differences in sampling of that study, which had recruited a lower proportion of the elderly. In contrast, the health severity perception of COVID-19 in this telephone survey was lower. This could be due to feeling fatigue with the epidemic news [42] or due to receiving more information about the situation and the virus, as the epidemic had evolved when compared with previous reported findings. Study findings indicated that respondents with chronic disease did not have higher levels of worry of getting COVID-19 when compared with those without chronic disease, even though the literature suggested that having chronic disease was considered as a risk factor for disease severity (complications and mortality) in COVID-19 [43,44,45,46]. Strengthening of information about those at higher risk in the community should be provided to raise the awareness of people at risk. Of note, the local population reported their belief that individuals (68.6%) bore comparable responsibility compared to the government (67.5%) to engage in infection control. Less than half of the respondents reported to have sufficient knowledge and accurate concepts to manage the health risk and safety during the outbreak [47]. For example, a quarter of the respondents did not perceive asymptomatic patients as infectious, which was similar to published findings in Egypt [48]. This misconception may affect the effectiveness of COVID-19 prevention efforts because the literature indicates that infected people can transmit the virus regardless of symptoms [7] and a high asymptomatic proportion of all infected patients was reported in Japan and Italy [6,49]. Furthermore, respondents with a misconception of the transmission route were found to be more likely to use television or radio as their main information source. As this misconception may likely be due to the lack of emphasis on asymptomatic patients as a transmission route, additional information related to the transmission route should be tailored to those with a lower education level.

Health-EDRM encompasses a wide range of components, where the component “Community Capacities for Health-EDRM” highlights the importance of the participation of the local population for managing the health risks in an emergency. Among the Health-EDRM behavioral practices [28], which are related to primary disease prevention practices [29], significant differences were found between attitude and practice in regards to washing hands with soap, ordering takeaways more often, wearing masks when going out, and avoiding going to COVID-19-confirmed regions outside Hong Kong. People who regarded these behaviors as useful tended to have a higher uptake rate. Consistent with Kwok et al. and Cowling et al. [38,41], the Hong Kong population showed a high compliance of wearing masks (94.2%), and there was a significant difference before and after the outbreak, which might be due to the high awareness of the outbreak, mass public health information announced through different channels, and the previous experience of SARS. However, more than half of respondents still reported traveling to COVID-19-confirmed regions before and during the study period. In addition, moderate and low uptake rates were found in using serving utensils (74.2%) and bringing their own utensils to meals (7.9%). Uptake patterns were found to be higher when compared with a previous study conducted during the H7N9 pandemic [27], where rates of 45.9% and 1.6%, respectively, were reported.

Meanwhile, no significant differences were found between perceived usefulness and practice of avoidance of dining or gathering together. This might suggest that even though people thought social avoidance might be useful to prevent COVID-19, they were unwilling to practice this socially limiting preventive behavior. As our study findings showed, males were less likely to avoid dining or gathering together, this might also provide the hypothesis that gender behavior might at least be associated with the gender imbalance in confirmed cases (with male predominance) at the beginning of the local outbreak [50]. In addition, “ordering takeaway food” was found to have increased by respondents during the epidemic. This increase may due to the social distancing promotion and regulations by the government (e.g., encouraging restaurants to provide takeaways as an option and avoiding table sharing) [51,52]. To promote better self-protection against infectious diseases, targeted interventions may focus on increasing the awareness towards the outbreak for the behaviors with a significant attitude–practice gap, while for the behaviors without an attitude–practice gap, additional measures to reinforce the practice were suggested. Further studies focusing on the barriers and self-efficacy might also be needed. Appropriate Health-EDRM health education and risk communication might wish to target subgroups who revealed suboptimal behavioral practices to further improve bottom-up response efforts.

Television, internet and smartphone apps were the top three channels for obtaining infectious disease information, covering more than 90% of the population. In Hong Kong, habits of television consumption have developed among the middle-aged and elderly, while the use of internet or smartphone apps were more popular in the younger age group. Consistent with the survey previously held in Hong Kong in February [41], almost all of the respondents would like to know more about the current situation of the outbreak, and how the government and themselves should respond. The availability and safety of COVID-19 vaccine were also of interest to general population.

Study limitations included the methodological limitations of telephone surveys. Firstly, households with no land-based telephone service may be missed. Nonetheless, the penetration rate of residential fixed line services in Hong Kong was 85.5% in December 2019 [53]. In addition, our study was conducted during the peak period of confirmed cases of COVID-19 in Hong Kong. During the data collection period, Hong Kong had 492 confirmed cases, accounting for almost half of the cases as of 14 April. Furthermore, the data collection occurred during the lockdown period in Hong Kong when people were advised to stay at home, and the government prohibited group gatherings of more than four people in public places under the Prohibition on Group Gathering Regulation. In addition, a landline telephone survey allows for more accurate geographic and demographic targeting when compared with mobile and online surveys. In general, specific subgroups, such as the elderly and the poor are more likely to be reached via landline than young adults [54]. Moreover, the landline telephone study methodology might capture populations that might be less technological or digital literate and it also offers the opportunity for researchers to compare with previous study findings with similar study methodology of other extreme events and disaster context. Finally, our sample was collected over a short period and comparable with the population census data in terms of age, gender, district of residence and marital status, which was generalizable to the Hong Kong population.

Secondly, the cross-sectional study design can only demonstrate associations between patterns and social-demographic predictors and causation cannot be attributed to the findings. A further cohort-based study design should be considered to monitor and assess changes in knowledge, attitude and practice patterns. Thirdly, this study might be subject to reporting bias since the information collected was self-reported, and data from non-respondents could not be obtained. In addition, the enquired personal or household health emergency disaster risks management (Health-EDRM) related behaviors and practices of COVID-19 prevention behavior were developed based on previous health and hygiene behavioral practices that might be relevant to contact transmission of the disease [33]. Yet, there are other relevant Health-EDRM behavioral practices in other cultural (non-Asia) and living contexts (e.g., rural community’s settings) that are worth exploring to generate evidence and develop health-risk education.

Last but not least, as the survey study was conducted after the implementation of the government preventive social distancing measures (e.g., restricting restaurant’s customer density, prohibition of group gathering), behavioral patterns might be subject to further changes if authorities have regulation and policy changes.

## 5. Conclusions

The Health-EDRM Framework is an integrated approach to manage health risks and build resilience. The COVID-19 pandemic offers an opportunity for the global community to understand how community and individuals might engage in disease prevention and health protection Health-EDRM behaviors that address a major biological hazard. The study findings indicated elderly and people with low education attainment had relatively poor knowledge and were less likely to adopt preventive Health-EDRM practices toward COVID-19. Tailored information with relevant information channels should be considered to reach these at-risk groups. Better understanding of uptake of knowledge, perception, attitude and behavior patterns by urban populations might facilitate better program and policy planning for Health-EDRM.

## Figures and Tables

**Table 1:** The respondent’s socioeconomic characteristics and comparison with Census population data.

Demographics	2016 Population Census		Study Sample		*p*-Value ^d^
Age	*n*	%	*n*	%	0.332
18–24	600,726	9.50%	71	9.30%	
25–44	2,228,566	35.26%	248	32.40%	
45–64	2,328,430	36.84%	303	39.60%	
65 or older	1,163,153	18.40%	143	18.70%	
Gender					0.425 ^e^
Male	2,850,731	45.10%	356	46.50%	
Female	3,470,144	54.90%	409	53.50%	
Marital status					0.962 ^e^
Non-married	2,523,742	39.93%	304	39.80%	
Married	3,797,133	60.07%	459	60.20%	
Residential district ^a,b^					0.334
Hong Kong Island	1,120,143	17.2%	147	19.20	
Kowloon	1,987,380	30.6%	231	30.20	
New Territory	3,397,499	52.2%	387	50.60	
Education ^a^					<0.001
Primary level or below	1,673,431	25.7%	61	8.00%	
Secondary	2,841,510	43.7%	330	43.30%	
Tertiary level	1,991,189	30.6%	371	48.70%	
Household Income ^c^					<0.001
<2000–7999	378,451	15.1	66	9.3	
8000–19999	649,450	25.9	101	14.1	
20000–39999	699,450	27.8	191	26.6	
40000 or more	782,383	31.2	360	50.2	

^a^ The Hong Kong population Census data additionally included age 15 to 17 years old. ^b^ Marine population was excluded. ^c^ The analysis was conducted with household data; only 718 households were available in our sample. ^d^ The χ^2^ test was used to measure the overall difference between this survey and the 2016 Hong Kong Population Census data. A *p*-value < 0.05 indicates a significant difference. ^e^ The χ^2^ test with continuity correction was used.

**Table 2:** Perceived usefulness and practice of preventive measures against COVID-19.

Control Measures that Can Protect from COVID-19 Infections	Thought It Was Useful for Prevention		Always or Usually Practicing Currently		Attitude vs. Practice ^a^
	*n*	%	*n*	%	*p*-Value
Wash hands before meals and after toilet	749	97.9	749	97.9	0.555 ^b^
Wash hands with soaps	740	96.7	706	92.3	<0.001
Wear mask when going out	753	98.4	745	97.4	<0.001 ^b^
Use serving utensil	708	92.5	568	74.2	0.174
Bring own utensils when dining out ^†^	542	81.9	52	7.9	0.199
Order takeaway more often	474	62.0	262	34.4	<0.001
Avoid dining or gathering together	742	97.0	616	85.0	0.178
Avoid going to public place or using public transport	713	93.4	408	53.5	0.002
Avoid going to COVID-19 confirmed regions outside Hong Kong	714	93.3	628	88.0	<0.001

^a^ Chi-square or Fisher’s exact test was used to test whether the perceived usefulness and practice are dependent. ^b^ Fisher’s exact test was used. ^†^ This analysis only included people who will go outdoors for a meal during the epidemic (*n* = 662).

**Table 3:** Factors affecting concern of getting COVID-19.

Characteristics		Not Worry or Don’t Know ^†^ (*n* = 252)	Worry (*n* = 505)	*p*-Value	AOR (95% CI)	*p*-Value
Age	18–24	15 (6.0%)	55 (10.9%)	0.037	1	
	25–44	77 (30.6%)	169 (33.5%)		0.567 (0.294–1.095)	0.091
	45–64	103 (40.9%)	198 (39.2%)		0.606 (0.318–1.153)	0.127
	65 or more	57 (22.6%)	83 (16.4%)		0.493 (0.244–0.995)	0.048
Gender	Male	132 (52.4%)	220 (43.6%)	0.022	1	
	Female	120 (47.6%)	285 (56.4%)		1.323 (0.956–1.831)	0.092
Chronic disease	No	206 (81.7%)	412 (81.6%)	0.957		
	Yes	46 (18.3%)	93 (18.3%)			
Education level	Primary level or below	25 (10.0%)	33 (6.6%)	0.249		
	Secondary level	108 (43.0%)	220 (43.7%)			
	Tertiary level	118 (47.0%)	250 (49.7%)			
Marital status	Non-married	99 (39.4%)	201 (39.9%)	0.908		
	Married	152 (60.2%)	303 (60.1%)			
Residential district	Hong Kong Island	52 (21.0%)	93 (18.4%)	0.207		
	Kowloon	83 (32.9%)	145 (28.7%)			
	New Territories	116 (46.0%)	267 (52.9%)			
Families members with chronic disease	No or don’t know	209 (82.9%)	393 (77.8%)	0.100		
	Yes	43 (17.1%)	112 (22.2%)			
Household floor area	350 ft or below	53 (21.6%)	100 (21.1%)	0.964		
	351 ft to 800 ft	157 (64.1%)	304 (64.0%)			
	801 ft or above	35 (14.3%)	71 (14.9%)			
Household income	<2000–7999	29 (12.1%)	36 (7.6%)	0.147		
	8000–19,999	36 (15.0%)	65 (13.8%)			
	20,000–39,999	66 (27.5%)	123 (26.1%)			
	40,000 or more	109 (45.4%)	248 (52.5%)			
Believing COVID-19 had large effect on their physical health	No	158 (62.9%)	206 (40.9%)	<0.001	1	
	Yes	93 (37.1%)	298 (59.1%)		1.583 (1.111–2.256)	0.011
Believing COVID-19 had large effect on their mental health	No	183 (72.6%)	219 (43.4%)	<0.001	1	
	Yes	69 (27.4%)	286 (56.6%)		2.490 (1.719–3.608)	<0.001
Believing COVID-19 had large effect on their financial status	No	181 (71.8%)	324 (64.2%)	0.035	1	
	Yes	71 (28.2%)	181 (35.8%)		0.927 (0.644–1.336)	0.685
Believing COVID-19 had large effect on their social life	No	104 (41.3%)	108 (21.4%)	<0.001	1	
	Yes	148 (58.7%)	397 (78.6%)		1.657 (1.138–2.413)	0.008
Believing COVID-19 had large effect on whole Hong Kong society	No	22 (8.7%)	20 (4.0%)	0.007	1	
	Yes	230 (91.3%)	485 (96.0%)		1.205 (0.608–2.385)	0.593
Perceived sufficient knowledge to manage COVID-19	No	127 (50.4%)	269 (53.3%)	0.456		
	Yes	125 (49.6%)	236 (43.7%)			
Perceived COVID-19 infectivity	Very low to medium or don’t know	16 (6.3%)	17 (3.4%)	0.058	1	
	High or very high	236 (93.7%)	488 (96.6%)		1.290 (0.610–2.728)	0.505
Perceived COVID-19 severity	Very low to medium or don’t know	58 (23.0%)	63 (19.0%)	0.197		
	High or very high	194 (77.0%)	409 (81.0%)			

^†^ 248 participants reported “not worried” and 4 reported “don’t know”.

## Appendix A

**Table A1:** Association between demographic variables and risk perception and knowledge about COVID-19.

Demographics	Risk Perception					Knowledge	
	Large Impact on Mental Health	Large Impact on Social Health	Large Impact on Financial Status	Large Impact on Hong Kong	Can be Prevented at Government Level	Can Be Spread by Insect	Can Be Spread by Asymptomatic Patient
Education							
Primary			1.10 (0.51–2.38)	0.15 (0.04–0.51) *		2.89 (1.35–6.18) *	0.45 (0.21–0.96) *
Secondary			1.81 (1.22–2.69) *	0.51 (0.21–1.23)		1.40 (0.87–2.26)	0.50 (0.32–0.78) *
Post-secondary			1	1		1	1
Gender							
Male	1	1					
Female	1.41 (1.02–1.95) *	1.60 (1.10–2.33) *					
Age							
18–24	2.00 (0.77–5.18)	6.12 (1.78–21.54) *	7.93 (2.76–22.76) *		3.06 (0.88–10.61)		
25–44	2.21 (1.19–4.12) *	4.03 (1.99–8.15) *	7.05 (3.35–14.81) *		2.12 (1.10–40.9) *		
45–64	1.26 (0.73–2.19)	2.98 (1.60–5.55) *	4.32 (2.22–8.42) *		0.96 (0.55–1.69)		
65+	1	1	1		1		
Household income							
<2000–7999			1				
8000–19,999			0.43 (0.20–0.92) *				
20,000–39,999			0.33 (0.16–0.72) *				
40,000 or more			0.25 (0.12–0.54) *				
Occupation							
White collar		1					
Blue collar		1.70 (0.99–2.93)					
Housewife		1.95 (0.98–3.90)					
Student		1.35 (0.36–5.05)					
Unemployment or retired		3.41 (1.72–6.76) *					

Note: Multivariable regression was not performed on outcome variables with a small number of cases (probability of that happening <0.05 or >0.95). Only variables with significant odds ratios in the multivariable logistic regression are shown in the table. None of these five demographic variables were significantly associated with believing COVID-19 had a large impact on their physical health, perceived sufficient knowledge of COVID-19, believing COVID-19 can be prevented at the household or individual level, perceived high infectivity and perceived severity of COVID-19. * indicates *p* < 0.05.

**Table A2:** Association between demographic variables and the practices (always or usually) of preventive measures against COVID-19.

Demographics	Wash Hands with Soaps	Avoid Dining or Gathering Together	Use Serving Utensil	Avoid Going to Public Place or Using Public Transport
Education				
Primary	0.31 (0.12–0.84) *		0.15 (0.04-0.51) *	
Secondary	0.68 (0.33–1.42)		0.51 (0.21–1.23)	
Post-secondary	1		1	
Gender				
Male	1	1		
Female	2.27 (1.17–4.43) *	1.82 (1.21–2.74) *		
Age				
18–24	2.15 (0.39–11.97)			2.48 (0.93–6.65)
25–44	4.62 (1.47–14.52) *			2.08 (1.09–3.98) *
45–64	2.83 (1.15–6.95) *			1.37 (0.77–2.44)
65+	1			1
Household income				
<2000–7999				
8000–19,999				
20,000–39,999				
40,000 or more				
Occupation				
White collar				1
Blue collar				0.70 (0.44–1.23)
Housewife				3.47 (1.87–6.42) *
Student				0.99 (0.37–2.65)
Unemployment or retired				2.29 (1.28–4.07) *

Note: Multivariable regression was not performed on outcome variables with a small number of cases (probability of that happening <0.05 or >0.95). Only variables with significant odds ratio in the multivariable logistic regression are shown in the table. None of these five demographic variables were significantly associated with bringing one’s own utensils when dining out, ordering takeaway food more often, and avoiding going to COVID-19-confirmed regions outside Hong Kong. * indicates *p* < 0.05.
